# Percutaneous dialysis arteriovenous fistula banding for flow reduction – a case series

**DOI:** 10.1186/s42155-018-0035-z

**Published:** 2018-11-03

**Authors:** Hong Kuan Kok, Julian Maingard, Hamed Asadi, Elizabeth Ryan, Mark Sheehan, Mark F. Given, Michael J. Lee

**Affiliations:** 1 Department of Interventional Radiology, Beaumont Hospital, Dublin, Ireland; 2 Department of Radiology, Royal College of Surgeons in Ireland, Dublin, Ireland; 3 Department of Interventional Radiology, Austin Hospital, Melbourne, Australia; 4 Interventional Neuroradiology Service, Austin Hospital, Melbourne, Australia; 5 School of Medicine, Faculty of Health, Deakin University, Geelong, VIC Australia

## Abstract

**Introduction:**

Arteriovenous fistulas (AVF) are the preferred method of vascular access for chronic haemodialysis. However, excess shunting through the AVF can result in dialysis-access steal syndrome (DASS) or high-output cardiac failure. Percutaneous AVF banding is a minimally-invasive technique for treating DASS with good short-intermediate term results.

**Materials and methods:**

We review a case series of percutaneous AVF banding procedures for DASS and high-output cardiac failure to illustrate the technique and limitations of this technique.

**Results:**

Two representative cases from our local experience were selected to illustrate the technique in a stepwise manner. Both cases were performed for DASS, with good technical success. However, clinical success was limited in one case due to underlying arterial insufficiency. The technique, selection of appropriate banding diameter for flow reduction, limitations and complications of alternative surgical techniques are discussed.

**Conclusions:**

Percutaneous AVF banding is a relatively straightforward and effective minimally-invasive technique for treatment of DASS supported by short-intermediate term data.

## Introduction

Arteriovenous fistulas (AVF) are the preferred method of vascular access for patients on chronic haemodialysis. Common AVF types encountered in practice include the radiocephalic, brachiocephalic and brachiobasilic fistulas (Vascular Access Work G 2006). Occasionally, synthetic polytetrafluoroethylene arteriovenous grafts (AVG) are utilised when there are no suitable veins to form an autologous conduit. Excess shunting through the AVF can result in dialysis-access steal syndrome (DASS) leading to distal ischaemia or high-output cardiac failure when severe. This risk is estimated to be approximately 1% in AVF and between 4 and 5% in AVG (Goel et al. 2006). Symptoms and signs of DASS may include pain, pallor, paraesthesia, weakness and necrosis of the distal extremity and digits in severe cases.

A number of treatment options are available for DASS and traditionally involve surgical procedures such as ligation, distal revascularisation and interval ligation (DRIL) and revision using distal inflow (RUDI) (Gupta et al. 2011). However, procedures such as surgical banding can potentially result in loss of vascular access for haemodialysis all together and the invasive nature of these procedures are associated with a relatively high rate of complications, ranging between 30 and 50% in one surgical series (Leake et al. 2015). Percutaneous endovascular banding, also known as Minimally Invasive Limited Ligation Endoluminal-assisted Revision (MILLER) has been described as a less-invasive fistula reduction technique for DASS (Goel et al. 2006), and in this article, we present a technical review of the procedure and discuss situations where percutaneous banding may have limited success.

## Materials and methods

We present two cases in which AVF banding was performed for DASS and high-output cardiac failure. Following written informed consent, all procedures were performed under aseptic technique, in a dedicated interventional radiology suite under conscious sedation and local anaesthesia.

## Results

### Case 1

**Fig. 1:**
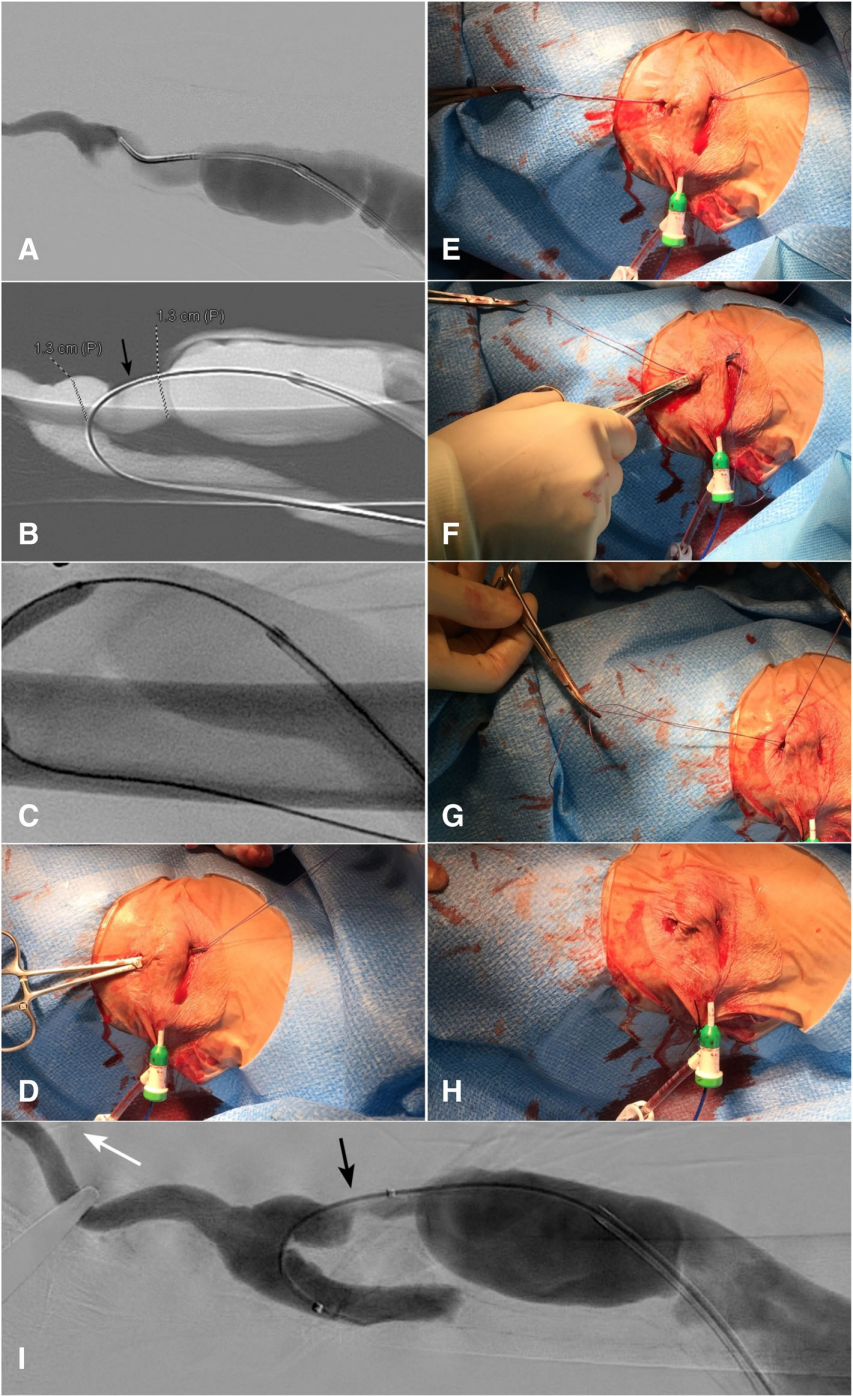
**a** Retrograde percutaneous access was obtained through the venous outflow limb of the brachiocephalic AVF and a 6-French sheath was placed. **b** A guidewire is negotiated across the anastomosis retrogradely into the inflow brachial artery and the juxta-anastomotic venous outflow limb diameter is measured at 13 mm (arrow). **c** Following this, a 5 × 40 mm angioplasty balloon was inflated to nominal pressure and (**d**) blunt dissection was performed carefully on either side of the venous outflow limb. **e-h** The venous outflow limb was stenosed to the profile of the inflated balloon using a constraining 2–0 suture. **i** Completion fistulogram showing stenosis in the outflow vein (black arrow) and improvement in perfusion of the brachial artery distal to the anastomosis (white arrow) as well as to the hand

A 50-year-old man on long term haemodialysis through a left-sided brachiocephalic AVF presented with worsening pain and cyanosis in the left hand. The AVF had also become progressively aneurysmal over the preceeding 2 months. Duplex ultrasound demonstrated non-occlusive laminated thrombus in the aneurysmal outflow vein. The distal radial and ulnar arterial pulses were weak with monophasic flow demonstrated on duplex assessment. A diagnostic fistulogram showed very rapid flow across the AVF with an aneurysmal outflow limb but no distal outflow or central venous stenosis (Fig. 1a). The juxtaanastomotic venous limb measured 13 mm in maximum diameter (Fig. 1b).

Percutaneous AVF banding was performed following ultrasound-guided retrograde access into the cephalic venous outflow limb with placement of a 6 French introducer sheath (Prelude; Merit Medical, South Jordan, UT). The brachial artery inflow was retrogradely catheterised using a 5 French angled KMP catheter (Cook, Bloomington, IN) and 0.035″ hydrophilic guidewire (Glidewire; Terumo, Somerset, NJ) combination. Following this, a 5 × 40 mm angioplasty balloon (EverCross; Covidien, Plymouth, MN) was placed across the venous outflow limb in the juxta-anastomotic region and inflated to nominal pressure using a mechanical inflation device (Fig. 1c). Two small incisions were made on either side of the inflated balloon and blunt dissection was carefully performed superficial and deep to the outflow vein (Fig. 1d).

A 2–0 braided absorbable suture (Vicryl; Ethicon, Somerville, NJ) was double looped, pulled through the incision, below and above the waist of the inflated angioplasty balloon and secured (Fig. 1e to h) to create a stenosis in the outflow vein (Fig. 1i). Immediate post-banding duplex ultrasound showed reduced velocities in the outflow vein. If there was no change on the immediate post-banding duplex study, the balloon can be exchanged for a smaller diameter balloon for creation of a smaller diameter band. In this case, there was complete resolution of DASS clinical symptoms and no issues related to dialysis or AVF thrombosis 6 months following the procedure.

### Case 2

**Fig. 2:**
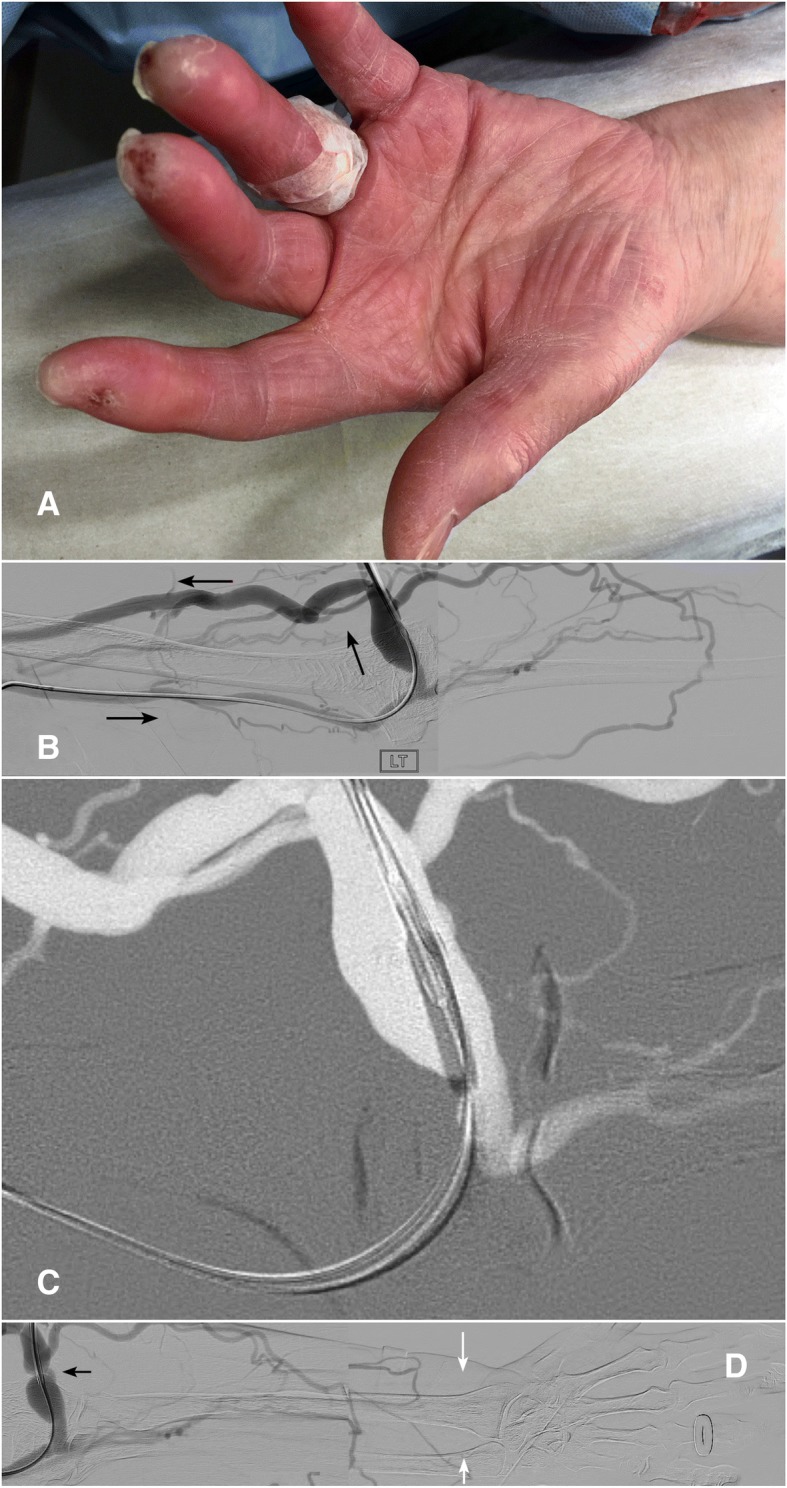
**a** Evidence of steal syndrome with associated tissue ulceration at the fingertips. **b** Diagnostic fistulogram performed through a catheter placed in the inflow brachial artery shows rapid shunting of flow across the AVF into the outflow cephalic vein (arrows indicating flow direction). The radial and ulnar arteries in the forearm are poorly opacified. **c** Percutaneous AVF banding was performed over a 5 × 40 mm angioplasty balloon with (**d**) a stenosis created in the outflow vein (black arrow). Perfusion to the forearm was improved but the distal radial and ulnar arteries (white arrows) were occluded secondary to underlying atherosclerosis and the perfusion to the hand did not improved significantly in this case

An 80-year-old female patient on long term haemodialysis due to diabetic nephropathy presented with a two-week history of left hand paraesthesia, weakness, ulceration and necrosis of her fingertips on the side of a left brachiocephalic AVF (Fig. 2a). The radial and ulnar arterial pulses were undetectable on clinical and duplex assessment. A diagnostic fistulogram was performed which demonstrated very brisk flow across the AVF with poor opacification of the radial and ulnar arteries in the forearm (Figs. 2b and c).

She proceeded to undergo percutaneous AVF banding using the same technique described in the case above (Figs. 2d and e). However, despite successful flow reduction through the AVF and some improvement of arterial flow to the level of the forearm, the distal perfusion to the hand remained poor due to underlying atherosclerosis with occlusion of the distal radial and ulnar arteries and absence of the palmar arch in the hand (Fig. 2f). The tissue loss was managed conservatively and eventually healed with associated soft tissue atrophy over a period of 8 weeks.

## Discussion

In patients with DASS, percutaneous AVF banding or MILLER is a useful and less-invasive alternative compared to surgical ligation where there is the possibility of loss of vascular access, requiring transition to dialysis catheters or pre-emptive creation of a new fistula. The technique for percutaneous AVF banding as described, is relatively straightforward and potentially reversible with angioplasty or stenting, if an absorbable suture is used as in our series (Shukla et al. 2016). Results from larger cohorts show promising results with a primary patency rate of between 75 and 85% at 6 months with primary assisted patency rates of 92% at 1 year (Shukla et al. 2016; Miller et al. 2010). Complications from percutaneous banding are uncommon and mostly relate to fistula thrombosis or need for repeat banding to achieve adequate clinical success (Shukla et al. 2016; Miller et al. 2010). Comparatively, surgical procedures including banding, DRIL and RUDI also demonstrate high fistula preservation rates between 89 and 100%, highest for DRIL procedures, but these were associated with a higher risk of early 30-day complications, particularly for surgical banding where there is an 11% risk of thrombosis and 2.8% infection risk in the early postoperative period (Leake et al. 2015).

The degree of juxtaanastomotic venous outflow reduction is determined by the luminal diameter reduction and is controlled by the diameter of the angioplasty balloon used. Miller and other authors aimed for a band size slightly less than the size of the downstream artery on the pre-reduction angiogram. We used a similar but slightly more conservative estimate in our cases, aiming for a balloon size under half of the juxtaanastomotic venous diameter as the relationship between the band stenosis and arterial diameter can change following redistribution of flow. The risks of AVF thrombosis is higher with greater reduction of flow and in our opinion, it is easier to repeat the banding procedure than salvage a thrombosed fistula. In the first case, the selection of a 5 mm balloon relative to a 13 mm segment resulted in an approximate angiographic stenosis of 75% (Fig. 1i). Banding can also be guided by intraprocedural flow measurements but these require additional flow transducer devices for measurement. Banding to a predetermined luminal reduction of approximately 75% appears to be safe, equally effective and reduces the need for additional costly equipment in most cases (Shukla et al. 2016).

Successful percutaneous AVF banding depends on the exclusion of other confounding causes for DASS symptoms, particularly arterial insufficiency as illustrated in the second case. Often, it can be difficult to confidently exclude distal arterial disease on non-invasive assessment because of the excess shunting and resultant poor distal perfusion prior to banding due to the small calibre of the distal palmar vessels and difficulties with optimal contrast timing on CT angiography. Therefore, upper limb digital subtraction angiography with treatment of underlying arterial stenoses prior to banding is essential for clinical success if there is clinical concern for associated arterial disease (Shukla et al. 2016). In particular, revascularisation strategies should aim to restore adequate inflow to the palmar arch and digits, particularly where tissue loss is of concern.

## Conclusion

Percutaneous AVF banding is an effective and safe minimally-invasive technique for treatment of ischaemic DASS with good intermediate-term patency rates, comparable to more invasive surgical procedures. However, there is a need for further studies to assess long-term outcomes including late thrombosis rates.

## Abbreviations

AVF: Arteriovenous fistula
AVG: Arteriovenous grafts
DASS: Dialysis-access steal syndrome
DRIL: Distal revascularisation and interval ligation
MILLER: Minimally Invasive Limited Ligation Endoluminal-assisted Revision
RUDI: Revision using distal inflow
